# Trajectory Adjustments Underlying Task-Specific Intermittent Force Behaviors and Muscular Rhythms

**DOI:** 10.1371/journal.pone.0074273

**Published:** 2013-09-30

**Authors:** Yi-Ching Chen, Yen-Ting Lin, Chien-Ting Huang, Chia-Li Shih, Zong-Ru Yang, Ing-Shiou Hwang

**Affiliations:** 1 School of Physical Therapy, Chung Shan Medical University, Taichung City, Taiwan; 2 Physical Therapy Room, Chung Shan Medical University Hospital, Taichung City, Taiwan; 3 Physical Education Office, Asian University, Taichung City, Taiwan; 4 Institute of Allied Health Sciences, College of Medicine, National Cheng Kung University, Tainan City, Taiwan; 5 Department of Physical Therapy, College of Medicine, National Cheng Kung University, Tainan City, Taiwan; University of Alberta, Canada

## Abstract

Force intermittency is one of the major causes of motor variability. Focusing on the dynamics of force intermittency, this study was undertaken to investigate how force trajectory is fine-tuned for static and dynamic force-tracking of a comparable physical load. Twenty-two healthy adults performed two unilateral resistance protocols (static force-tracking at 75% maximal effort and dynamic force-tracking in the range of 50%–100% maximal effort) using the left hand. The electromyographic activity and force profile of the designated hand were monitored. Gripping force was off-line decomposed into a primary movement spectrally identical to the target motion and a force intermittency profile containing numerous force pulses. The results showed that dynamic force-tracking exhibited greater intermittency amplitude and force pulse but a smaller amplitude ratio of primary movement to force intermittency than static force-tracking. Multi-scale entropy analysis revealed that force intermittency during dynamic force-tracking was more complex on a low time scale but more regular on a high time scale than that of static force-tracking. Together with task-dependent force intermittency properties, dynamic force-tracking exhibited a smaller 8–12 Hz muscular oscillation but a more potentiated muscular oscillation at 35–50 Hz than static force-tracking. In conclusion, force intermittency reflects differing trajectory controls for static and dynamic force-tracking. The target goal of dynamic tracking is achieved through trajectory adjustments that are more intricate and more frequent than those of static tracking, pertaining to differing organizations and functioning of muscular oscillations in the alpha and gamma bands.

## Introduction

Visuomotor tracking is a critical function of the motor system. However, intrinsic trajectory control is affected by variations in the state of the motor system [1], [2], since motor responses are not strictly smooth. A larger size of force variability greatly drifts the force output away from an intended priori standard. The complexity of force variability is another dimension of force variability [3], [4], typically indexed with entropy measures [3], [5] to characterize the degree of fluctuation predictability over a force data stream [6], [7]. The size and the complexity of force variability of a visuomotor task can be differently organized. For instance, tracking with visual feedback is more accurate and has a smaller size but a greater complexity of force variability than tracking without visual feedback [1], [3], [8]. An increase in force complexity is related to engagement of trajectory adjustments using on-line sensory inputs, rather than to task degradation [1], [9]. One of the major sources of force or kinematic variability comes from sampled feedback processes of the visuomotor system [10], [11] for enhancing the stability of the visuomotor system against long feedback delays [10], [12]. However, sampled feedback brings about movement intermittency, as manifested with discrete blocks of pulse-like elements in movement trajectory. Movement intermittency becomes less evident in pursuit of a predictable target [13], [14] or removal of visual feedback [10]. Both kinematic and force profiles exhibit intermittency, which is related to internal coding of the planned trajectory and error correction [14], [15].

Exertion level is a key factor of force variability underlying progressive recruitment of fast-twitch motor units [16] and variations in code rating [17]. On account of an exertion-dependent increase in force variability [18], [19], precise control of force is far more difficult at a higher force range than at a lower force range. Force stability at a higher force range presumably relies on task-dependent variations in code rating in that motor units are largely recruited [20]. As movement accuracy at large force output is insufficient for precision tasks, force scaling at a higher force range is often overlooked. Little attention has been paid to contrasting force variability properties between static and dynamic force-tracking at relatively high exertion levels. It is apparent that static and dynamic force-tracking challenge the visuomotor system to different extents, including visual information load [21], proprioceptive inputs [22], target constraints to produce the criterion force [23], and so on.

The present study sought to contrast the size and complexity of force intermittent behaviors between static and dynamic force-tracking at relatively high exertion levels of equivalent physical loads. Because of the different time and target constraints, we expected intermittent force behavior and the scaling property of individual force pulse for the two force-tracking tasks to be task-dependent. Another focus of this study was to explain the task-dependent intermittent force behavior with oscillatory activities in the working muscle. It was hypothesized that, in comparison to static force-tracking, dynamic force-tracking would lead to larger force intermittency and a smaller amplitude ratio of the a priori standard of intended pursuit relative to force intermittency, greater complexity and spectral dispersion of the force intermittency profile, and greater force pulse metrics with different statistical properties. In addition, muscular oscillations during static and dynamic force-tracking were differently organized with respect to tracking protocols. Our observations on force intermittency dynamics and muscular oscillations extend previous work to gain better insight into how force trajectories are planned to satisfy differing task needs.

## Methods

### Ethics Statement

The research project was approved by an authorized institutional human research review board (Chung Shan Medical University Hospital Institutional Review Board, CSMUH IRB), and all subjects signed informed consents before the experiment, conforming to the Declaration of Helsinki.

### Subjects

Twenty-two male subjects (mean: 21.6±1.2 years) from a local community and a university participated in this study. All of the subjects were self-reported as being right-handed, and none of them had symptoms or signs of neuromuscular diseases.

### Experiment Procedures

This study employed two unilateral resistance protocols of gripping, static and dynamic force-tracking. Each protocol consisted of three trials of 20 seconds, which were randomly completed by our participants with inter-trial periods of rest of at least 3 minutes. The subject sat on a chair with the left arm hanging naturally by the trunk and gripped a hand dynamometer (sensitivity: 0.01 N, bandwidth: DC–1 kHz, Model 9810P, Aikoh, Japan) connected to an analog amplifier (Model: PS-30A-1, Entran, UK). The force output and the target curve were displayed on a computer monitor to guide the force exertion of the force-tracking maneuver. Before the experiment, all subjects first performed 3 maximal voluntary contractions (MVC) of 3 seconds, separated by 3-minute pauses. The mean of the maximal force for the 3 MVCs was defined as the peak gripping force. During static force-tracking, the subjects needed to produce a constant force of 75% of peak gripping force with the aid of visual feedback. Dynamic force-tracking required the subjects to exert a load-varying isometric force to couple a 0.5 Hz sinusoidal target wave in the range of 50%–100% of peak gripping force. The target signal moved vertically in a range of 7.2° of visual angle (i.e., 3.6° above and 3.6° below the eye level on the screen), and visual feedback gain in terms of visual angle per MVC was identical for static and dynamic force-tracking. Muscle activity of the left flexor digitorum superficialis (FDS) was recorded by surface electromyography. A bipolar surface electrode unit (1.1 cm in diameter, gain  = 365, CMRR  = 102 dB, Imoed Inc., USA) was placed at an oblique angle approximately 4 cm above the wrist on the palpable muscle mass. All signals were sampled at 1 kHz by an analog-to-digital converter with 16-bit resolution (DAQ Card-6024E; National Instruments Inc., Austin, TX, USA), controlled by a custom program on a Labview platform (Labview v.8.5, National Instruments Inc., Austin, TX, USA).

### Data Processing

#### The size and complexity of the force intermittency profile

**Figure 1:**
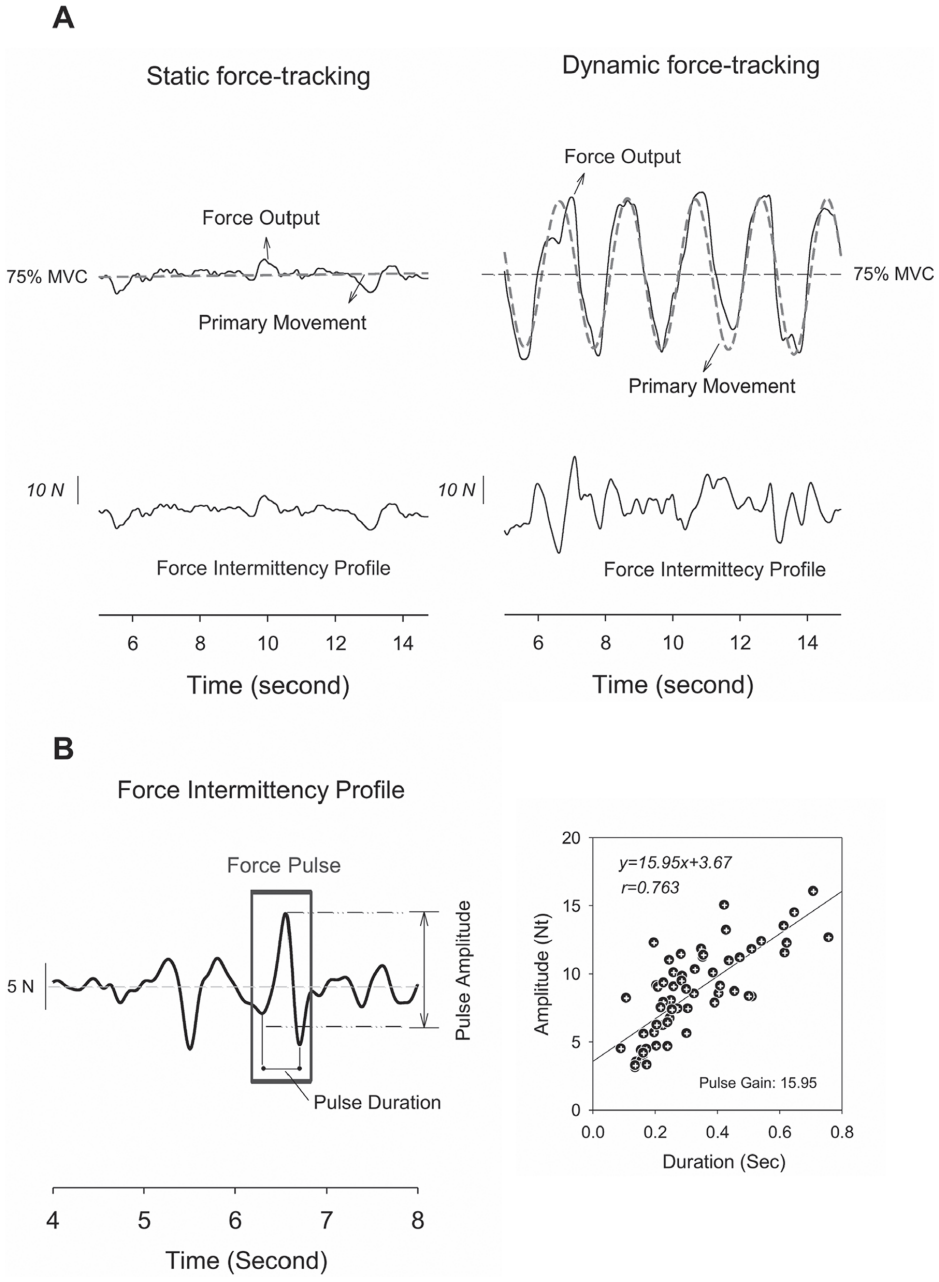
Illustrative examples of force intermittency profile, primary movement, and force pulse. (A) Feature extraction of force intermittency profile and primary movements from force outputs of static and dynamic force-tracking. (B) Representative force intermittency profile during dynamic and static tasks.

Gripping force was down-sampled to 100 Hz in off-line analysis and then conditioned with a low-pass filter (cut-off frequency: 6 Hz) [24]. Mean gripping forces of an experimental trial for both force-tracking paradigms were determined. Then force output of the tracking tasks was dichotomized into two different force components, primary movement and force intermittency profile, akin to the algorithms proposed by Roitmen et al. (2004) and Selen et al. (2006) [25], [26]. In brief, the primary movement was a smooth and deterministic force component of the force-tracking task, spectrally identical to the target rate. Also, the primary movement approximated target movement in amplitude. Therefore, the primary movement symbolizes the a priori standard of intended pursuit to couple the target signal. On the other hand, the force intermittency profile was a stochastic force component that contributed to force variability. The force intermittency profile was irregular, containing a number of individual force pulses (Fig. 1A). Recent studies have validated that force pulses are not noises, but part of an additive accuracy control to remedy tracking deviations from the target trajectory [26], [27]. The dichotomy of gripping force was helpful to specify structural changes in the force intermittency profile (force variability) and to differentiate task effects on deterministic and stochastic force components for static and dynamic tracking. For the static force-tracking, the primary movement was a force level of 75% MVC. The force intermittency profile of static force-tracking could be obtained by removing the linear trend of the force time series (Fig. 1A, left). For the dynamic task, the primary movement was a 0.5 Hz sinusoidal wave with amplitude roughly in the range of 50%–100% MVC. The force intermittency profile of dynamic force-tracking was obtained by conditioning the force output with a zero-phasing notch filter that passes all frequencies except for a target rate at 0.5 Hz (Fig. 1A, right). The transfer function of the notch filter was 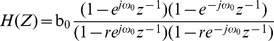, *r* = .9975, *ω*_0_ = *π*/360. Subtracting the force intermittency profile from the dynamic force output gave the sinusoidal component of the target rate in the gripping force, previously described as the primary movement for the dynamic task.

Root mean square (RMS) was applied to the primary movement and the force intermittency profile to calculate the amplitudes of the two force components. The RMS of the force intermittency profile symbolized the size of force variability. The amplitude ratio of the primary movement to force intermittency (R_PM/FI_) was defined as the RMS of the primary movement divided by the RMS of the force intermittency profile. Spectral distribution of the force intermittency profile was estimated with the Welch method and a fast Fourier transform with a spectral resolution of 0.1 Hz. Mean frequency and spectral dispersion (spectral ranges between the 10th and 90th percentiles of the power spectra) were determined from the force intermittency spectral profile. The complexity of the force intermittency profile (i.e., the complexity of force variability) was quantified with multi-scale entropy (MSE) to reveal a sample entropy (SampEn) curve across different time scales (Appendix S1) [6], [28]. Each time scale represented 10 ms for the sampling rate of 100 Hz. MSE areas under the time scales 1–25 (or 10–250 ms) and 26–60 (or 260–600 ms) were empirically determined to measure the complexity of the force intermittency profile on short and high time scales, respectively. The MSE area of the overall time scale of 1–60 was the sum of MSE areas under the time scales 1–25 and 26–60. A higher MSE area indicated a noisier structure with greater signal complexity.

#### Force pulse variables

Individual force pulses in a force intermittency profile were identified afterwards. Local peak in the force intermittency profile was defined as a force pulse, and a force intermittency profile contained many force pulses. Amplitude of each force pulse was the difference between a local maximum and the average value of the two nearest minima (Fig. 1B, left) [24], [25]. The pulse duration was the time between two successive local minima in the force intermittency profile. For each subject, we characterized the pulse amplitude and duration of each pulse in a force intermittency profile during static and dynamic force-tracking and then calculated the probability distribution of pulse amplitude and pulse duration to get mean pulse amplitude and mean pulse duration. Linear regression between the pulse duration and pulse amplitude in a force intermittency profile provided a duration-amplitude regression slope, or pulse gain (Fig 1B, right) [24], [25]. Force pulse gain of the three experiment trials during static and dynamic force-tracking was averaged across the subjects.

#### EMG variables

EMG of the FDS muscles were conditioned with band-pass filters (pass band for EMG: 1∼400 Hz). The amplitude of the EMG of the FDS muscles for the entire period of a trial was represented with RMS. The EMG data after band-pass filtering were rectified for spectral analysis [29]. Rectification of surface EMG is believed to enhance the spectral peaks that symbolize common oscillatory inputs or the mean firing rate of an active muscle [30], [31], [32]. The power spectra of the both un-rectified and rectified EMG signals were computed using Welch's method. A Hanning window with a window length of 1.6 seconds and an overlap of 0.4 seconds was used. Spectral resolution was 0.244 Hz. The spectral profile of rectified EMG of the three trials was averaged and then normalized with the mean spectral amplitude to reduce population variability. We obtained mean spectral peaks in the alpha (8–12 Hz), and gamma (35–50 Hz) bands from three tracking trials during static and dynamic force-tracking. All signal processing was completed using Matlab (Mathworks Inc., Natick, MA, USA).

#### Statistical Analysis

For each subject, all force and EMG variables of the three trials were averaged for the static and dynamic force-tracking tasks. A paired t-test was used to compare the mean gripping force between static and dynamic force-tracking. Hotelling's T^2^ test was used to contrast the population means of force intermittency properties between static and dynamic force-tracking, including the amplitude parameter of force intermittency (RMS values of primary movement/force intermittency and R_PM/FI_), spectral parameters of force intermittency (mean frequency and spectral dispersion), complexity of force intermittency (MSE areas in short, long, and overall time scales), scaling of force pulses (pulse amplitude, pulse duration, and pulse gain), and EMG variables (alpha peak and gamma peak, and RMS) of the FDS muscle. Post-hoc analysis was conducted for all Hotelling's T^2^ tests with Bonferroni correction to determine the significance levels for multiple comparisons. For both tracking conditions, the correlation between the force amplitude variables (RMS__PM_, RMS__FI_, and R_PM/FI_) and standardized amplitude of spectral peaks was examined with Pearson's correlation. Likewise, the correlation between the force intermittency complexity (MSE areas in low and high time scales) and standardized amplitude of spectral peaks was also examined with Pearson's correlation. The levels of significance for the determination of differences were 0.05. All statistical analyses were completed with the statistical package for Social Sciences (SPSS) for Windows v. 15.0 (SPSS Inc., USA).

## Results

### Basic Force Characteristics

The results of paired t statistics suggested an insignificant protocol effect on mean gripping force between dynamic force-tracking (128.79±6.05 N) and static force-tracking (130.89±6.11 N) (*t_21_* = −1.471, *P* = 0.156), which validated that the physical work of the two loaded paradigms was very similar.

### Force Intermittency Properties and Force Pulse Metrics

**Figure 2:**
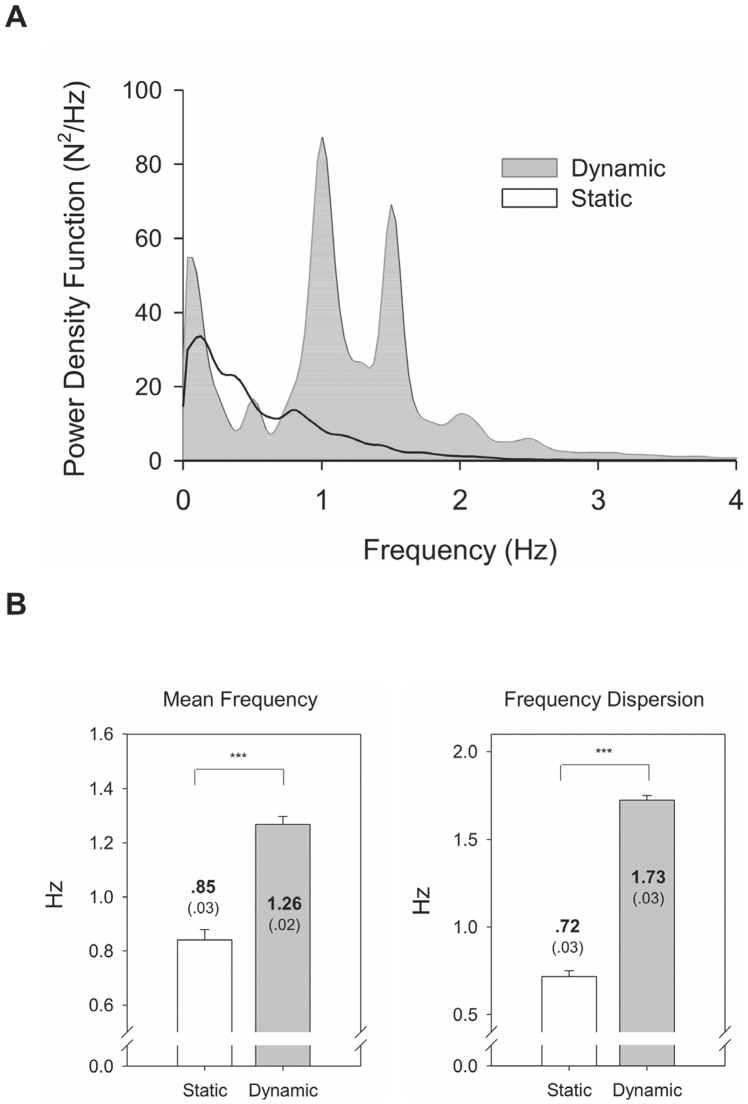
Contrast of spectral features of force intermittency profile between static and dynamic force-tracking. (A) Pooled spectral distributions of force intermittency profile during static and dynamic force-tracking, (B) population means of mean frequency and spectral dispersion for force intermittency profiles (Post-hoc test: ^***^: Dynamic > Static, *P*<.001).

**Figure 3:**
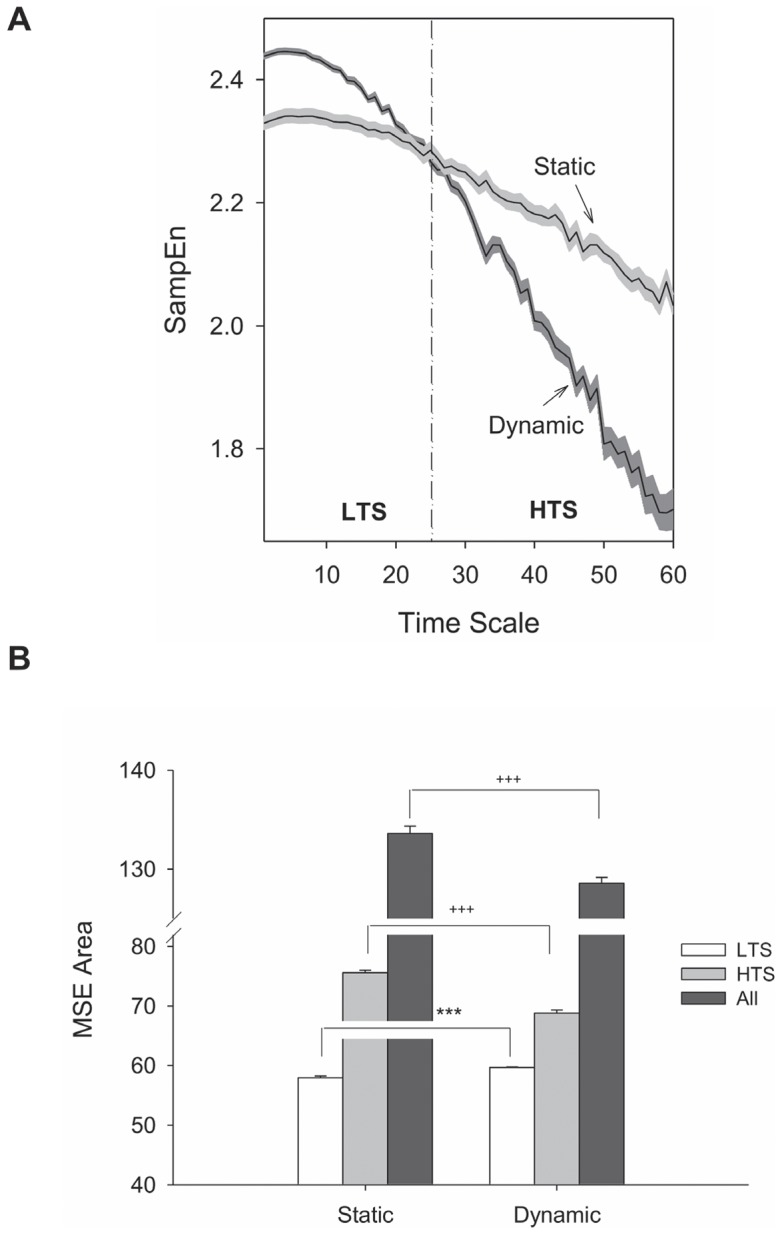
Contrasts of pooled complexity measures of force intermittency profile between static and dynamic force-tracking. (A) Sample entropy (SampEn) versus time scales, (B) Multi-scale entropy area (MSE area) for the low time scale of 1–25 (LTS), high time scale of 26–60 (HTS), and overall time scale of 1–60 (All). Each time scale represents 10 ms due to the sampling rate of 100 Hz. (Post-hoc test: ^***^: Dynamic > Static, *P*≦.001; ^†††^: Static > Dynamic, *P*<.001).

Table 1 contrasts the mean amplitudes for the primary movement (PM) and force intermittency (FI) profile between static and dynamic tracking. Hotelling's T^2^ suggested a significant protocol effect on RMS values of the primary movement and force intermittency profile, as well as the amplitude ratio of R_PM/FI_ (Wilks' Λ  = 032, *P*<.001). Post-hoc analysis revealed that the RMS value of the force intermittency profile during dynamic force-tracking was greater than that during static force-tracking (*P*<.001), whereas the RMS value of the primary movement did not differ between the two force-tracking conditions (*P* = .301). Static force-tracking exhibited a greater R_PM/FI_ (48.01±3.49) than did dynamic force-tracking (22.52±0.51) (*P*<.001). Figure 2A shows the power spectra of force intermittency between static and dynamic force-tracking for all subjects. The mean frequency and spectral dispersion of the force intermittency profile differed with force-tracking mode (Wilks' Λ  = .035, *P*<.001), with greater mean frequency and spectral dispersion for dynamic force-tracking (*P*<.001) (Fig. 2B). Figure 3A shows the results of MSE analysis and pooled SampEn curves across different time scales for static and dynamic tracking. Dynamic force-tracking appeared to exhibit a larger SampEn in the low time scale 1–25 but a smaller SampEn in the high time scale 26–60 than those of static force-tracking. Hotelling's T^2^ and post-hoc analysis were consistent with that observation (Wilks' Λ  = .106, *P*<.001). Dynamic force-tracking had a larger MES area (59.7±0.3) under the time scale 1–25 than did static force-tracking (57.9±0.3) (*P* = .001), but an opposite trend was noted for the MES area under the time scale 26–60 (Dynamic: 68.8±0.5; Static: 75.6±0.5) (*P*<.001) (Fig. 3B). The MES area of the overall time scale 1–60 for dynamic tracking (128.6±0.6) was significantly lower than that for static force-tracking (133.6±0.7) (*P*<.001) (Fig. 3B), because of a more potent effect on the deceasing trend of the MES area in the high time scale.

**Table 1:** The contrast of amplitude variables of the primary movement and force intermittency between static and dynamic tracking.

Amplitude variable^1^	Static	Dynamic	*Statistics*
**RMS__PM_ (N)** 3	126.23±4.97	124.19±4.36	
**RMS__FI_ (N)** 4	3.49±0.32	5.68±0.26^***^	Λ = 0.032, *P* = .000 2
**R_PM/FI_** 5	48.01±3.49^†††^	22.52±0.51	

1 Values were presented as mean ± se.

2 Post-hoc for static force-tracking vs. dynamic force-tracking (^***^: Dynamic > Static, *P*<.001; ^†††^: Static > Dynamic, *P*<.001).

3 RMS__PM_: root mean square of primary movement.

4 RMS__FI_: root mean square of force intermittency profile.

5 R_PM/FI_ denotes amplitude ratio of the primary movement to force intermittency.

The fundamental element in the force intermittency profile was the force pulse, the scaling parameters of which were examined between static and dynamic force-tracking (Table 2). Hotelling's T^2^ statistics showed that the pulse variables differed with tracking protocol (Wilks' Λ  = .135, *P*<.001). Post-hoc analysis suggested that the pulse amplitude of dynamic force-tracking (9.88±.53 N) was larger than that of static force-tracking (3.30±.35 N) (*P*<.001). Dynamic force-tracking exhibited a longer pulse duration (.448±.007 sec) than did static force-tracking (.378±.110 sec) (*P*<.001). The pulse gain (amplitude-duration regression slope) of dynamic force-tracking (26.59±1.39 N/sec) was significantly greater than that of static force-tracking (11.78±1.17 N/sec) (*P*<.001).

**Table 2:** The contrast of force pulse variables between static and dynamic tracking.

Force pulse variable^1^	Static	Dynamic	*Statistics*
**Mean Amplitude (N)**	3.30±.35	9.88±.53^***^	
**Mean Duration (Sec)**	.378±.011	.448±.007^***^	Λ = 0.135, *P* = .000 2
**Pulse Gain** 3 ** (N/Sec)**	11.78±1.17	26.59±1.39^***^	

1 Values were presented as mean ± se.

2 Post-hoc for static force-tracking vs. dynamic force-tracking (^***^: Dynamic > Static, *P*<.001).

3 Pulse gain also denotes amplitude-duration slope of force pulse.

### EMG Variables and Muscular Oscillations

**Figure 4:**
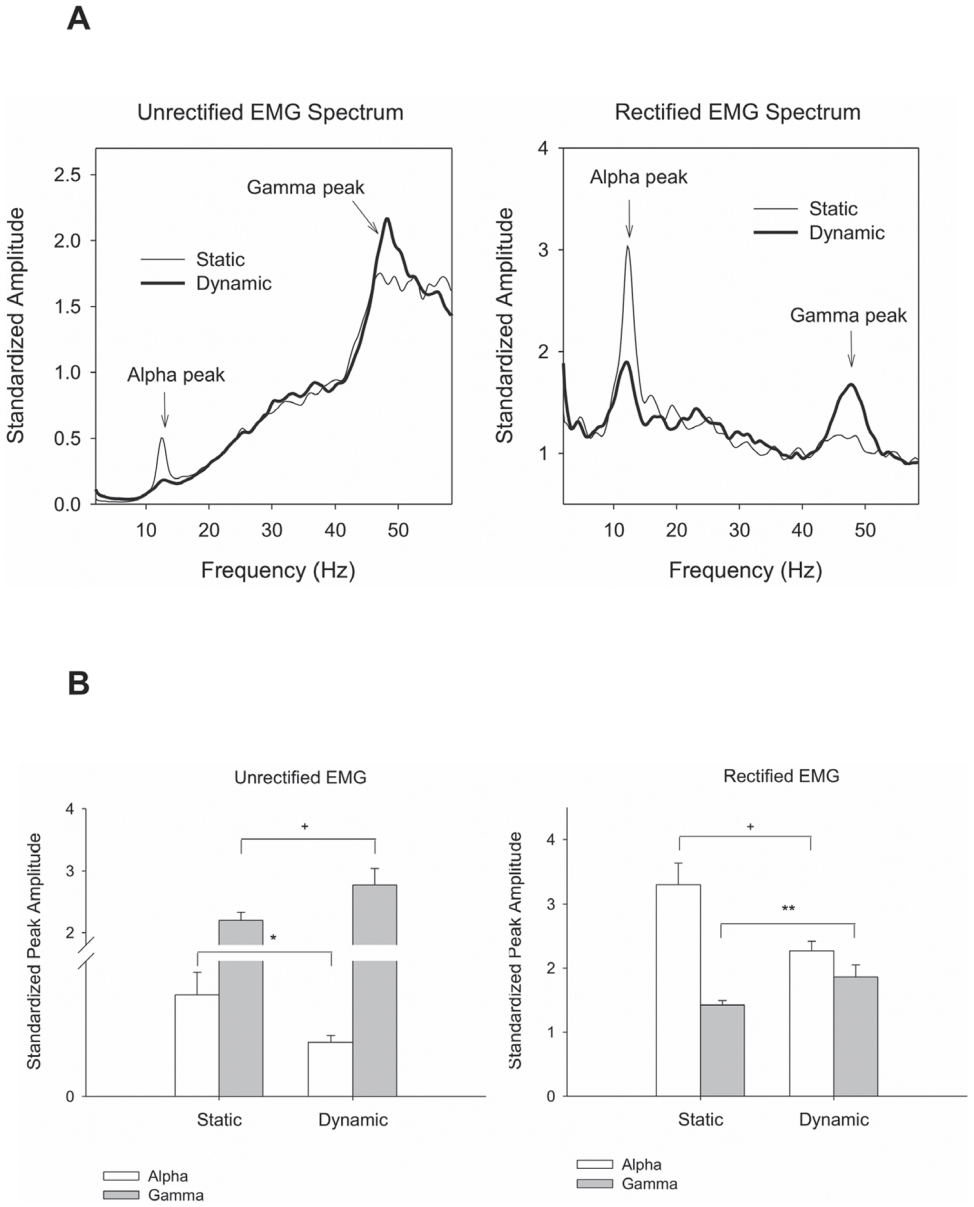
Contrasts of spectral features of the EMG between static and dynamic force-tracking. (A) Pooled spectral profiles of un-rectified and rectified EMG, (B) The means and standard errors of standardized amplitude for 8–12 Hz and 35–50 Hz spectral peaks. (Post-hoc test: ^*^: Dynamic > Static, *P*<.05; ^††^: Static > Dynamic, *P*<.01;^ †^: Static > Dynamic, *P*<.05).

Figure 4A contrasts the pooled spectral profiles of un-rectified/rectified EMG of the FDS muscle between static and dynamic force-tracking. Both EMG spectral profiles exhibited two prominent spectral peaks in 8–12 Hz and 35–50 Hz. Hotelling's T^2^ statistics showed that EMG spectral variables varied with force-tracking protocol (Un-rectified EMG: Wilks' Λ  = .722, *P* = .039; Rectified EMG: Wilks' Λ  = .496, *P* = .003) (Fig. 4B). For rectified EMG, post-hoc analysis further revealed that static force-tracking (normalized spectral amplitude: 3.30±0.34) had a greater alpha spectral peak (8–12 Hz) than dynamic force-tracking (2.27±0.15) (*P* = .009). Conversely, the dynamic task (standardized amplitude: 1.86±0.19) exhibited a larger gamma rhythm (35–50 Hz) than the static task (standardized amplitude: 1.42±0.07) (*P* = .011). Variations in standardized spectral peaks in the alpha and gamma bands between static and dynamic gripping for un-rectified EMG were similar to those of rectified EMG. Static gripping resulted in a greater alpha peak but a smaller gamma peak (alpha: 0.45±0.10; gamma: 2.20±0.13) than dynamic gripping (alpha: 0.24±0.03; gamma: 2.77±0.27) (*P*<.05). However, the EMG RMS of the FDS muscle was not significantly different between dynamic (0.065±0.005 mV) and static force-tracking (0.064±0.005 mV) (*P* = .829).

Table 3 shows relationships between the force intermittency variables and muscular oscillations for static and dynamic force-tracking. For static force-tracking, the standardized amplitude of the 8–12 Hz spectral peak was not significantly related to any force intermittency variables (*P*>.05). For dynamic force-tracking, the standardized amplitude of 35–50 Hz spectral peak was also correlated negatively and positively with force intermittency amplitude (*P*<.05) and the amplitude ratio of R_PM/FI_ (*P*<.001), respectively. However, the standardized amplitude of the 8–12 Hz spectral peak was independent of any force variables (*P*>.05), though the muscular oscillation was significantly suppressed in comparison with that during static force-tracking.

**Table 3:** Pearson's correlation coefficients between force intermittency characteristics and muscular oscillations.

	Static	Dynamic	
(n = 22)	Alpha	Alpha	Gamma
**RMS__PM_** 1	r = −.336, *P* = .126	r = −.289, *P* = .192	r = −.223, *P* = .319
**RMS__FI_** 2	r = –.365, *P* = .095	r = –.208, *P* = .354	r = –.426, *P* = .048^*^ 7
**R_PM/FI_** 3	r = .382, *P* = .079	r = –.381, *P* = .081	r = .654, *P* = .001^**^ 7
**MSE__LTS_** 4	r = .052, *P* = .189	r = .282, *P* = .204	r = .295, *P* = .183
**MSE__HTS_** 5	r = .118, *P* = .602	r = –.104, *P* = .645	r = .057, *P* = .800
**MSE__All_** 6	r = .088, *P* = .698	r = –.031, *P* = .892	r = .089, *P* = .695

1 RMS__PM_ represents root mean square of primary movement.

2 RMS__FI_ represents root mean square of force intermittency profile.

3 R_PM/FI_ represents amplitude ration of primary movement relative to force intermittency profile.

4 MSE__LTS_ represents multi-scale entropy area of low time scale 1–25.

5 MSE__HTS_ represents multi-scale entropy area of high time scale 26–60.

6 MSE__All_ represents multi-scale entropy area of overall time scale 1–60.

7 The shaded area indicates a significant level of correlation coefficient. (^*^: *P*<.05;^ **^: *P*<.005).

## Discussion

The present study first revealed that the size and complexity of force intermittency as well as muscular oscillation were organized with the target goal of the force-tracking tasks. The dynamic force-tracking brought about a greater size of force intermittency with higher and wider spectral dispersion than did static force-tracking. In comparison with static tracking, dynamic tracking exhibited a greater complexity of force intermittency in the low time scale but, conversely, a greater regularity of force intermittency in the high time scale. Concurrent with task-dependent scaling of force intermittency, dynamic force-tracking exhibited a more potentiated 35–50 Hz muscular oscillation but a smaller 8–12 Hz muscular oscillation than did static force-tracking. In light of the force intermittency and muscular rhythm, there exist strategic differences in force regulation between dynamic and static force-tracking of a comparable load along with an underlying greater cognitive challenge for repetitive transient force changes during dynamic force-tracking.

### Trajectory Optimization and Task-dependent Force Intermittency Properties

In this study, force output during a tracking maneuver was dichotomized into two force components, the smooth primary movement and the force intermittency profile. Contrary to a primary movement that signifies a priori standard preprogrammed in pursuit of a visual target [24], [25], [27], the force intermittency profile reflects an error-correction strategy in an attempt to remedy deviations during goal-directed movement. Under the framework of sampled movement control [10], [11], [12], force pulses in a force intermittency profile are centrally-scalable, superimposed onto the primary movement to tune a force trajectory [24], [26], [27]. Since dynamic tracking produced larger force intermittency and a smaller R_PM/FI_ ratio than did static force-tracking (Table 1), dynamic force-tracking weighs more heavily on the error-correction process, entailing more intensive integration of proprioceptive and visual inputs than does static tracking [33]. Also, corrective adjustments to dynamic force-tracking were more frequent in order to generate motor commands in shorter time scales, on account of the higher number of high-frequency components with greater spectral dispersion in the force intermittency profile (Figs. 2A, 2B). Irrespective of static and dynamic tracking, force intermittency had a spectral range under 2 Hz, consistent with the Vaillancourt et al. (2002) [34], who reported a 0–2 Hz dominant frequency in force output during static continuous isometric contraction with low and high visual gains. Interestingly, force intermittency during dynamic force-tracking appeared to oscillate at harmonics of the target rates (primarily 1.0 Hz, 1.5 Hz, and 2 Hz). It is speculated that the subjects recurrently updated the trajectory control at particular rates, which have been noted to code kinematic properties of repetitive hand movement in the cortico-cerebello-cortical loop [35], [36].

Although the complexity of force intermittency is typically characterized with approximate entropy [5], [7], [37] or uni-scale SampEn [23], this study adopted a new complexity measure with the use of multi-scale entropy (MSE). The methodological advantage of using MSE is that it allows assessment of SampEn across multiple time scales on the basis of multiple coarse-grained sequences and long-range temporal correlations, such that MSE accounts for time-dependent complexity and the presence of memory effects in physiological data [6], [28]. In the low time scale 1–25, dynamic force-tracking exhibited a greater force intermittency complexity (larger MSE area) than did static force-tracking (Figs. 3A, 3B), physically in accordance with the wider spectral spreads in high frequency of the force intermittency profile. Dynamic force-tracking in the shorter time scale was more informative, probably because the force tracking system adapted the required force output to multiple changing sensory inputs from the periphery to remedy tracking deviations in a short interval [33]. However, force intermittency of dynamic force-tracking in the high time scale 26–60 were conversely more regular (smaller MSE area) than those of static force-tracking (Figs. 3A, 3B). Since the target cycle of 0.5 Hz for dynamic force-tracking was 500 ms, it was very likely that the force intermittency data in the former half of the target cycle shared some stochastic properties with the latter half of the target cycle. Therefore, the force intermittency sequence after the course-gaining process with a window length exceeding half of the target cycle (time scale  = 25) presented memory effects with higher possibility of predictability (lower SampEn curve) than did the force intermittency sequence in the static condition. This scenario suggests that fine-tuning of force trajectory during dynamic tracking was rhythmically encoded in every half a target cycle. The trajectory corrective mode for time-to-valley force and time-to-peak force during dynamic force-tracking could be analogous. Because the effect of SampEn in the high time scale on complexity measures overpowered that in the low time scale, the overall MSE area of dynamic force-tracking was still lower than that of static force-tracking (Fig. 3B). This observation on overall MSE area can explain a more regular movement trajectory for tracking a periodically-moving target [5], [23]. Like force intermittency properties, force pulse metrics were differently organized with target accuracy constraints. Dynamic tracking exhibited greater pulse amplitude and pulse duration than did static tracking (Table 2). In addition, to keep in line with a rhythmic target movement, the central nervous system had to multiply pulse gain (or scaling amplitude-duration slope) during dynamic tracking (Table 2). Therefore, the dynamic target goal was accomplished by additive accuracy control that preferentially increased the gain of spatial scaling of force pulse more than the gain of temporal scaling of force pulse. A similar change in scaling amplitude-duration slope of kinematic submovement was reported, when tracking speed progressively increased during circular manual tracking [24], [25].

### Oscillatory Muscular Activity and Task-dependent Trajectory Adjustments

The variations in force intermittency property for the static and dynamic force-tracking pertained to differing organization of muscular oscillations at 8–12 Hz and 35–50 Hz in the FDS muscle (Fig. 4A). Research has shown that muscular oscillations in the EMG spectral peaks are related to grouped motor unit firing rates, especially enhanced EMG rectification that suppresses EMG spectral features related to the motor unit action potential shape (higher-frequency components) [31], [38]. Although we did not directly measure the EEG-EMG piper rhythm (EMG-EEG coherence), it is likely that the muscular oscillations at 8–12 Hz and 35–50 Hz were physiological tremor [39], [40] and the gamma band of the EMG piper rhythm [1], [41], respectively. They could be the peripheral parts of EEG-EMG piper rhythm serving to regulate motor unit firing during force tracking maneuvers. For dynamic force-tracking, the most noteworthy finding was the potentiation of the low gamma band of the EMG piper rhythm (Fig. 4B). In fact, oscillatory muscle activity in 35–50 Hz is in line with converging evidence that the gamma band in corticomuscular coherence presents during phasic movement [1], [42] and repetitive isotonic contraction [43]. The occurrence of gamma synchrony is thought to be of functional relevance when a motor task entails temporal modulation in movement patterns with global alertness to integrate sensory-motor information [1], [42], [44]. Our observation adds to this hypothesis by showing a significant negative correlation between 35–50 Hz muscular oscillation and force intermittency amplitude (Table 3). Hence, we may well argue that the gamma EMG piper rhythm is specified for fine-tuning force trajectory during dynamic tracking. The more gamma EMG piper rhythm associates with the lesser corrective attempts and the greater priori standard of tracking maneuver relative to force intermittency (R_PM/FI_). In addition, we noted a significant suppression of alpha muscular oscillation during dynamic force-tracking, as compared with that of static tracking (Fig. 4B). Iyer et al. [45] also reported a roughly 12 Hz motor unit discharge during static and quasi-sinusoidal isometric contraction at the same mean force level. However, what is still not completely clear is the role of 8–12 Hz muscular oscillatory activity in the shift of tracking mode in this study.

In this study, muscular rhythm was assessed with spectral peaks of surface EMG. EMG rectification is a prevailing approach prior to calculating corticomuscular coherence for maximizing information about the grouped firing rate frequencies of active motor units [30]–[32]. However, some researchers argue against the appropriateness of the pre-processing procedure, as rectified EMG does not necessarily enhance the peak detection of corticomuscular coherence and may produce inconsistent coherence spectra in some cases [46], [47]. Regarding this methodological controversy, we also validated our observations with spectral analysis using raw EMG. Two prominent spectral peaks were consistently noted in the spectral profiles of raw and rectified EMG, with similar parametric changes in standardized peak amplitude with respect contraction mode. In addition, we did not observe a significant EMG oscillation in the beta range (13–21 Hz) in either profile during static force-gripping, though previous studies have shown that the beta EMG-EEG piper rhythm is critical to maintaining force stability during sustained isometric contraction [1], [29], [42]. Physically, an evident EMG-EEG coherence in the beta band just represents a relatively high degree of an in-phase oscillation at 13–21 Hz for both EEG and EMG signals; however, it does not mean a prominent beta oscillation as compared to other spectral ingredients in the EMG signal. Despite this fact, future work is still needed to find cortical control over the task-specific scaling of force intermittency force-tracking of different patterns, on account of the functional interactions between cortical and spinal oscillatory networks.

## Conclusions

In light of characteristic differences in the primary movement and force intermittency, we noted that neuro-mechanic control of force trajectory for static and dynamic force-tracking at a relatively high exertion level was task-dependent. Dynamic force-tracking exhibits a greater amount of force intermittency, with higher spectral components and greater complexity in the low time scale than that of static force-tracking. The target goal of dynamic force-tracking is achieved through frequent and vast trajectory adjustments, underlying intricate short-term and similar error-correction processes over half a target cycle. Unlike during static force-tracking, alpha muscular oscillation is markedly suppressed during dynamic force-tracking. The emergence of gamma muscular oscillation during dynamic force-tracking is likely to be responsible for the scaling of force intermittency and force trajectory adjustments. At a relatively high exertion level, modulations of muscular oscillation and force intermittency properties agree with the theoretical postulation that internal force coding to stabilize movement trajectory differs vastly with target constraints.

## Supporting Information

Appendix S1 **Calculation of MSE Area.**
(DOCX)
