# Supplementary material for: Trajectory Adjustments Underlying Task-Specific Intermittent Force Behaviors and Muscular Rhythms
Source: PLoS One. 2013 Sep 30;8(9):e74273. doi: 10.1371/journal.pone.0074273 (PMC3787025; doi:10.1371/journal.pone.0074273)
Supplement: Appendix S1 — Calculation of MSE Area. (DOCX) [file pone.0074273.s001.docx]

**Appendix SA: Calculation of MSE Area**

The calculation of the multi-scale entropy area (MSE Area) consists of three steps. (Ref. 6,28)

**The first step:** Obtain the coarse-grained sequences of the down-sampled submovement trace {*X^(τ^* ^)^}

 (a)

where {z_1_, z_2_, . . ., z_N_} is the time series of submovement and τ is the time scale

**The second step:** Calculate SampEn for each coarse-grained sequence {*X^(τ^* ^)^}

MSE^(τ )^ = {SampEn_(τ )_,_τ=1,2,…60_} (b)

SampEn measures the negative natural logarithm of an estimate of the conditional probability that epochs of length m that match point-wise within a tolerance level (r) also match at the next point. Here, r = 20% of the standard deviations of X(τ ), and m = 2. (Ref. 6)

**The third step:** Sum each MSE^(τ )^ across different time scales (τ)

$MSE area of low-time scale=\sum_{\tau=1}^{25} \mathrm{SampEn}$ (c)

$MSE area of high-time scale=\sum_{\tau=25}^{60} \mathrm{SampEn}$ (d)
